# Isolation and Genomic Characterization of Avian Reovirus From Wild Birds in South Korea

**DOI:** 10.3389/fvets.2022.794934

**Published:** 2022-01-28

**Authors:** Sang-Won Kim, Yu-Ri Choi, Jong-Yeol Park, Bai Wei, Ke Shang, Jun-Feng Zhang, Hyung-Kwan Jang, Se-Yeoun Cha, Min Kang

**Affiliations:** Department of Veterinary Infectious Diseases and Avian Diseases, Center for Poultry Diseases Control, College of Veterinary Medicine, Jeonbuk National University, Iksan, South Korea

**Keywords:** avian reoviruses, wild bird, chicken, South Korea, σC-encoding gene, σNS-encoding genes

## Abstract

Avian reoviruses (ARVs) cause severe arthritis, tenosynovitis, pericarditis, and depressed growth in chickens, and these conditions have become increasingly frequent in recent years. Studies on the role of wild birds in the epidemiology of ARVs are insufficient. This study provides information about currently circulating ARVs in wild birds by gene detection using diagnostic RT-PCR, virus isolation, and genomic characterization. In this study, we isolated and identified 10 ARV isolates from 7,390 wild birds' fecal samples, including migratory bird species (bean goose, Eurasian teal, Indian spot-billed duck, and mallard duck) from 2015 to 2019 in South Korea. On comparing the amino acid sequences of the σC-encoding gene, most isolates, except A18-13, shared higher sequence similarity with the commercial vaccine isolate S1133 and Chinese isolates. However, the A18-13 isolate is similar to live attenuated vaccine av-S1133 and vaccine break isolates (SD09-1, LN09-1, and GX110116). For the p10- and p17-encoding genes, all isolates have identical fusion associated small transmembrane (FAST) protein and nuclear localization signal (SNL) motif to chicken-origin ARVs. Phylogenetic analysis of the amino acid sequences of the σC-encoding gene revealed that all isolates were belonged to genotypic cluster I. For the p10- and p17-encoding genes, the nucleotide sequences of all isolates indicated close relationship with commercial vaccine isolate S1133 and Chinese isolates. For the σNS-encoding gene, the nucleotide sequences of all isolates indicated close relationship with the Californian chicken-origin isolate K1600657 and belonged to chicken-origin ARV cluster. Our data indicates that wild birds ARVs were derived from the chicken farms. This finding suggests that wild birds serve as natural carriers of such viruses for domestic poultry.

## Introduction

Avian reoviruses (ARVs), classified under the Orthoreovirus genus of *Reoviridae* family, have a non-enveloped icosahedral double capsid with 10 double-stranded RNA (dsRNA) genome segments which are further divided into three size classes based on their electrophoretic mobility: large (L1, L2, L3), medium (M1, M2, M3), and small (S1, S2, S3, S4) (1). ARV genome encodes eight structural (λA, λB, λC, μA, μB, σA, σB, and σC) and four non-structural viral proteins (μNS, σNS, p10, and p17) (2). Among the S-class segments of ARV, the segment S1 contains three open reading frames (ORFs) that are translated into p10, p17, and σC proteins (2). The p10 protein induces cell fusion (3), while the p17 protein induces cellular protein translation shut-off and cell cycle arrest (4, 5). The σC protein encoded by the third ORF of the segment S1 is the cell attachment protein and elicits the production of ARV-specific neutralizing antibodies (6, 7). The σC sequence is used as a genetic marker to characterize and classify ARV isolates into different six or seven genotypic clusters (8, 9). The σNS protein encoded by the segment S4, has been reported for its single-stranded RNA (ssRNA) binding activity, and it is divided into diverse lineages that conserved by host origin of ARVs (10, 11); as well it has been used for diagnostic analysis (10, 12).

ARVs are widespread in nature and affect various commercial and wild avian species. ARVs have been isolated from poultry, such as chickens, ducks, turkeys, ostriches, and wild birds (13–17). Major damages from ARV infections are observed in young chickens, and ARV infections in broiler chickens are associated with up to 10% mortality and up to 40% morbidity. ARV infections in broilers have been associated with various clinical diseases, such as viral arthritis/tenosynovitis, malabsorption syndrome (MAS), runting–stunting syndrome (RSS), respiratory diseases, hepatitis, myocarditis, neurological signs (incoordination, tremors, and twisted necks), and immunosuppression (18, 19). ARV infections are responsible for significant economic losses in the poultry industry (20). There have been ARV infection outbreaks in the USA, Canada, Brazil, Europe, Israel, China, and Korea (8, 21–26).

ARVs have also been identified in wild birds, such as crows, magpies, partridges, black-capped chickadees, brown-eared bulbuls, psittacine bird species, and mallard ducks (27–35). ARV infections in wild birds have been associated with mortality and/or various clinical diseases, such as hepatitis, enteritidis, and neurological signs (29, 31, 33, 34, 36, 37). A previous study on the seroprevalence of ARVs in wild birds reported 25% seroprevalence in bean goose (*Anser fabalis*) and 34% seroprevalence in white-fronted goose *(Anser albifrons frontalis*) in Germany in 1998 (38). In addition, antigenic prevalence in wild birds representing 32 species was reportedly ~30% in Poland from 2014 to 2016 (12). ARVs have been isolated from hooded crows in Finland, American crows in the USA, brown-eared bulbuls in Japan, psittacine bird species in Germany, and mallard ducks in China (27, 29, 30, 34, 35). Various hypotheses have been proposed about the role of wild birds in the dissemination and maintenance of ARVs and pathogenic potential of ARVs in wild birds that usually commingle around poultry farms. In previous studies, ARVs isolated from wild birds were somewhat distant from the those from chicken farms (29, 30, 33–35). However, recently research showed that the σA-encoding gene isolated from a healthy ostrich at a domestic farm in Japan showed great similarity to the chicken-origin ARVs, and the σC-encoding gene isolated from magpies was found to be genetically similar to the chicken-origin ARVs (13, 31). This genetic association between isolates from wild birds and poultry can be explained by the potential of wild birds to act as a carrier for the transmission of the ARVs (31). However, studies on the role of wild birds in the epidemiology of ARVs are insufficient.

This study provides information about currently circulating ARVs in wild birds by gene detection using diagnostic RT-PCR, virus isolation, and genomic characterization of S1 and S4 gene. Furthermore, it investigates the possibility of wild birds being potential sources of infection in poultry.

## Materials and Methods

### Sample

A total of 7,390 fresh fecal samples were obtained from major migratory bird habitats, including near commercial/domestic chicken premises in South Korea by an avian influenza virus national monitoring program (2015–2019). All live migratory birds were trapped and collected feces by the South Korea Animal and Plant Quarantine Agency (QIA) and the Ministry of Agriculture, Food and Rural Affairs (MAFRA) (39). Ten fecal samples obtained from pure or separately standing flocks of the same species were pooled into one test sample. Finally, the 739 resultant test samples were 10-fold diluted in sterile phosphate-buffered saline [PBS, pH7.4; supplemented with 100X antibiotic–antimycotic (Gibco, New York, USA)], thoroughly mixed by vortexing, and centrifugated at 600 x *g* for 10 min at 4°C. The supernatant was subsequently filtered (0.45-μm pore size; Minisart® NML, Sartorious, Germany). The filtered supernatant was then conserved in aliquots at −70°C for virus isolation and viral RNA extraction.

### RNA Extraction and RT-PCR

Viral RNA was extracted from the clarified fecal samples using the MagMAX^TM^ - 96 AI/ND Viral RNA isolation kit (Thermo Fisher Scientific, Vilnius, Lithuania) with KingFisher Duo Prime Purification system (Thermo Fisher Scientific, Waltham, MA) following the manufacturer's protocol. Viral cDNA was generated from RNA samples using GoScript^TM^ reverse transcriptase (Promega, Madison, WI USA) with random primers (9-mers; TaKaRa Bio. Inc., Otsu, Shiga, Japan). In the reverse transcription (RT) reaction, 8 μL of extracted RNA and 2 μL of dimethyl sulfoxide (DMSO, Tedia, USA) were heated at 100°C for 5 min and then placed in an ice bath for 5 min. Then, the following were added to this reaction mixture: 8 μL of GoScript^TM^ 5X RT reaction buffer (Promega, Madison, WI USA), 10 μL of 2.0 mM of each dNTP (SolGent, Daejeon, Korea), 4 μL of MgCl_2_ (Promega, Charbonnie‘re, France), 1 μL of 20 units Recombinant RNasin® Ribonuclease Inhibitor (Promega, Madison, WI, USA), 1 μL of GoScript^TM^ reverse transcriptase, 1 μL of 50 pmol random primer, and 4 μL of diethylpyrocarbonate-treated water (DEPC water; Biosesang, Seoul, Korea); a final volume of 39 μL was obtained. The RT reaction mixture was incubated in this sequence: 25°C for 5 min, 42°C for 60 min, and 70°C for 15 min to inactivate the enzyme. The cDNA amplification was performed in 50 μL volume, containing 10X Taq buffer (Solgent, Daejeon, Korea), 5U Taq DNA polymerase (Solgent, Daejeon, Korea), 10 pmol of each primer (Table 1). For the detection of ARVs in wild bird feces, partial S1 gene was amplified. Thermal cycling protocols were as follows: Initial denaturation at 94°C for 5 min, 35 cycles (denaturation at 94°C for 1 min, annealing at 50°C for 1 min, extension at 72°C for 1 min) and one final extension at 72°C for 10 min (40). For the sequence analysis, viral RNA was extracted from virus stocks using the Viral Gene-SpinTM Viral DNA/RNA Extraction kit (iNtRON, Daejeon, Korea). Viral cDNA was obtained from RNA samples by the above-mentioned method. For the amplification of the full S1 gene, thermal cycling protocols were as follows: Initial denaturation at 94°C for 5 min, 40 cycles (denaturation at 94°C for 1 min, annealing at 60°C for 1 min, extension at 72°C for 100 s) and one final extension at 72°C for 10 min (2). For the amplification of the full S4 gene, thermal cycling protocols were as follows: Initial denaturation at 94°C for 5 min, 35 cycles (denaturation at 94°C for 30 s, annealing at 58°C for 30 s, extension at 72°C for 90 s) and one final extension at 72°C for 10 min (41).

**Table 1:** RT-PCR primer sequences and expected PCR products.

**Designation**	**Sequence (5'-3')**	**Gene**	**Location**	**PCR products (bp)**	**Reference**
MK87	5'-GGTGCGACTGCTGTATTTGGTAAC-3'	Partial S1	55–78	532	(40)
MK88	5'-AATGGAACGATAGCGTGTGGG-3'	Partial S1	568–588		
ARV-S1-1632-F	5'-CAATCCCTTGTTCGTCGATGYT-3'	Full S1	8–28	1,632	(2)
ARV-S1-1632-R	5'-AATAACCAATCCCMGTACGGCG-3'	Full S1	1,618–1,639		
SnS-F	5'-CTTTTTGAGTCCTTGTGCAGCCAT-3'	Full S4	2–26	1,185	(41)
SnS-R	5'-TAAGAGTCCAAGTCGCGGCAGAGG-3'	Full S4	1,163–1,186		

### DNA Barcoding for Species Identification of Fecal Samples

The host species of the PCR-positive fecal samples were identified using the DNA barcoding technique as previously described (42). Briefly, DNA was extracted from fresh fecal samples using an Accuprep® Stool DNA Extraction kit (Bioneer, Daejeon, Korea) according to the manufacturer's protocol. The primers Aves-F (5′-GCATGAGCAGGAATAGTTGG-3′) and Aves-R (5′-AAGATGTAGACTTCTGGGTG-3′) were used to amplify the mitochondrial cytochrome oxidase gene subunit I present in host feces (43). PCR products were sequenced and identified using the information available at the Barcode of Life Data Systems website (Biodiversity Institute of Ontario, University of Guelph, Guelph, Ontario, Canada; http://www.barcodinglife.org/views/login.php) (44).

### Virus Isolation

The PCR-positive samples were inoculated into the yolk sac of 6-day-old specific pathogen-free (SPF) embryos (SPAFAS Poultry Company, Jinan, China). Embryonated eggs were candled daily for 5 days. Chorioallantoic membranes (CAMs) of the infected embryos were collected by three cycles of freezing and thawing; then, 1.5 mL PBS was added, followed by centrifugation at 6,000 x *g* for 10 min. For cell culture passage, chicken embryo liver (CEL) cells were prepared from 14-day-old SPF chicken embryos as per the standard protocol and then dispensed into a 6-well-cell culture plate (SPL life sciences, Pocheon, Korea). The medium for CEL cell culture was Eagle's minimum essential medium supplemented with 8% fetal bovine serum (FBS) and 1% addition of 100X antibiotic–antimycotic. When the cell monolayers were ~80% confluent, the medium was aspirated. Cell monolayers were infected with 0.2 mL of 10-fold diluted CAM fluids from the first passage of chicken embryos and incubated at 37°C for 60 min. Then, a maintenance medium containing 4% FBS was added. The cultures were incubated at 37°C under 5% CO_2_ and were observed daily under a microscope to check for a cytopathic effect (CPE). Once 70–80% CPE was developed, the cultures were subjected to three cycles of freezing and thawing and then clarified with low centrifugation at 600 x *g* for 20 min (41).

### Cloning and Sequencing

PCR products of full-length S1 and S4 genes were cloned and sequenced. The PCR products of the expected lengths were purified with the QIAquick gel extraction kit (Qiagen, Chatsworth, CA) and then cloned into the pGEM-T easy vector (Promega, Madison, WI, USA) according to the manufacturer's instructions, and nucleotide sequences were determined using an ABI 3730XL DNA Analyzer (Applied Biosystems, Foster City, CA) by SolGent (Daegeon, Korea) (45).

### Sequence Analysis and Phylogenetic Analysis

Based on the nucleotide and deduced amino acid sequences of the σC- and σNS-encoding genes, phylogenetic analysis was carried out. Sequence alignments and pairwise sequence comparisons were performed using the GENETYX software (Genetyx Corp., Tokyo, Japan) (46). Phylogenetic analysis was performed using the maximum likelihood method of MEGA-X software package (version 10.0.5) with bootstrap values calculated from 1,000 replicates (47). Recombination detection was accomplished by using Bootscan analysis within the Simplot program version 3.5.1, using the neighbor-joining method, with a Kimura 2-parameter applied and 100 replicates (48).

## Results

### Virus Isolation and Propagation

**Figure 1:**
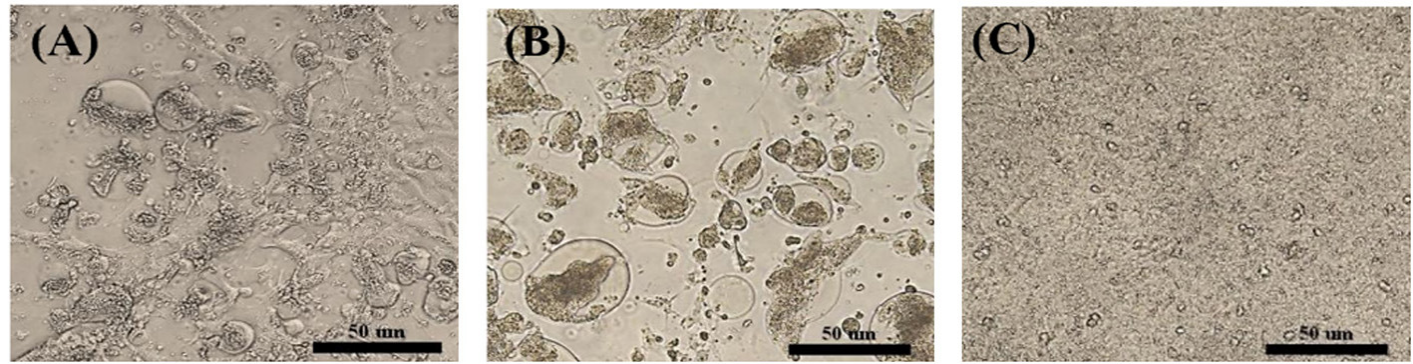
Cytopathic effect (CPE) in ARV-infected CEL cells. **(A)** S1133/Chicken/USA/1971 infected CEL cells forming a syncytial cluster at 1 day post infection. **(B)** Representative wild bird isolate (A18-13/Wild bird/Korea/2018) infected CEL cells forming a syncytial cluster at 1 day post infection. **(C)** Non-infected CEL cell control.

A total of 14 PCR-positive samples from 739 samples (1.9%) were identified. Ten ARVs were isolated by using CEL cell from the 14 PCR-positive samples, and all isolates were confirmed by diagnostic RT-PCR as described above in Table 1. Five ARVs were detected from bean goose (*Anser fabalis*), one from mallard duck (*Anas platyrhtchos*), one from Eurasian teal (*Anas crecca*), one from oriental turtle dove (*Streptopelia orientalis*), and one from Indian spot-billed duck (*Anas poecilorhyncha*); the source could not be identified for one ARV (Table 2). CPE was observed in CEL cell infected with the 10 isolates. The CPE of isolates manifested as detachment of the monolayer or syncytium formation (Figure 1).

**Table 2:** ARV obtained in the study, origin of sample, year of isolation, host species, sample type, and GenBank accession number.

**No**.	**Isolate**	**Province**	**Year**	**Host**	**Sample type**	**GenBank accession number**	
						**S1**	**S4**
1	A15-19	Jeonnam	2015	Not detected	Fecal	MW357863	MW357853
2	A15-48	Jeonnam	2015	Bean goose (*Anser fabalis*)	Fecal	MW357864	MW357854
3	A15-71	Jeonbuk	2015	Bean goose (*Anser fabalis*)	Fecal	MW357865	MW357855
4	A15-108	Jeonbuk	2015	Bean goose (*Anser fabalis*)	Fecal	MW357866	MW357856
5	A15-113	Jeonnam	2015	Bean goose (*Anser fabalis*)	Fecal	MW357867	MW357857
6	A15-157	Jeonbuk	2015	Oriental turtle dove (*Streptopelia orientalis*)	Fecal	MW357868	MW357858
7	A18-13	Gyeongnam	2018	Eurasian teal (*Anas crecca*)	Fecal	MW357869	MW357859
8	A18-19	Jeonnam	2018	Indian spot-billed duck (*Anas poecilorhyncha*)	Fecal	MW357870	MW357860
9	A18-205	Jeonnam	2018	Mallard (*Anas platyrhynchos*)	Fecal	MW357871	MW357861
10	A19-106	Gyeongnam	2019	Bean goose (*Anser fabalis*)	Fecal	MW357872	MW357862

### Sequence Analysis

The full-length S1 and S4 genes of all ARV isolates were sequenced, and the obtained sequences were submitted to the GenBank database under the accession numbers showed in Table 2. The σC- and σNS-encoding genes of all isolates were analyzed and compared with reference isolates (Supplementary Table 1). The alignment of nucleotide sequences of the σC-encoding gene of 10 isolates showed high similarity with commercial vaccine isolates (av-S1133, S1133, 2408, and 1733) (98.4–99.9%) and Chinese chicken-origin isolates (GuangxiR1, SD09-1, LN09-1, and GX110116) (98.6–99.6%) in nucleotide sequence. Conversely, the 10 isolates showed low similarity with previously identified Korean chicken-origin isolates (SNU0044, SNU0046, K738-14, and iREO0309) (50.2–74.5%) and Tvärminne avian virus (TVAV)-like wild bird isolates (Pycno-1, Corvus corone cornix 2002, and Tvärminne avian virus) (48.5–53.9%) (Supplementary Table 2). An alignment of nucleotide sequences of the σNS-encoding gene of the 10 isolates showed high similarity with the Californian chicken-origin isolate K1600657 (92.7%−92.9%) and low similarity with the TVAV-like wild bird isolates (Pycno-1, SD-12, Tvärminne avian virus, 71-03, Chickadee, and SRK) (58.2%−77.7%) (Supplementary Table 3).

We compared the amino acid sequences of the 10 isolates with those of 15 reference isolates. The results were found to be consistent with the results of pairwise comparisons of the σC-encoding gene (Supplementary Table 4). Compared at six conservative amino acid mutations (24, 71, 106, 113, 134, and 135), most newly isolated viruses, except A18-13 isolate, shared higher sequence similarity with commercial vaccine isolate S1133 and Chinese chicken-origin isolates (GuangxiR1 and GuangxiR2). Whereas 10 isolates shared lower similarity with Korean chicken-origin isolates (SNU0044, SNU0046, iREO0309, and K738-14). Nucleotide changes in the σC ORF unique to A18-13 isolate and the live attenuated vaccine av-S1133 and vaccine break isolates (SD09-1, LN09-1, and GX110116) were identified. A18-13 isolate, live attenuated vaccine, and vaccine break isolates carry four nonsynonymous nucleotide substitutions (A^71^, G^317^, T^338^, and C^405^), whereas commercial vaccine isolate S1133 have nucleotide substitutions at C^71^, C^317^, C^338^, and A^405^ in the σC-encoding gene (Supplementary Table 4). These four non-synonymous nucleotide substitutions lead to deduced amino acid substitutions (T24N, T106R, T113I, and V135I) in the σC-encoding gene (Table 3). Nucleotide changes in the σNS ORF unique to all isolates and the Californian chicken-origin isolate K1600657 were identified. All isolates and K1600657 carry 10 non-synonymous nucleotide substitutions (G^215^, T^361^, A^455^, A^472^, G^478^, G^484^, T^652^, A^760^, G^817^, and C^1049^), whereas commercial vaccine isolates, including live attenuated vaccine have nucleotide substitutions at A^215^, C^361^, G^455^, G^472^, A^478^, C^484^, G^652^, C^760^, A^817^, and T^1049^ in the σNS-encoding gene (Supplementary Table 4). These 10 non-synonymous nucleotide substitutions lead to deduced amino acid substitutions (D72G, P121S, R152H, A158T, S160D, P162A, A218S, L254T, T273V, and M350T) in the σNS-encoding gene (Table 3). For the p10 protein, all isolates were not observed for deduced amino acid substitutions in putative transmembrane domains. For the p17 protein, all isolates were not observed for deduced amino acid substitutions in nuclear localization signal (NLS) motif (Supplementary Table 5).

**Table 3:** Deduced amino acid substitutions in the σC- and σNS-encoding genes of ARV isolates from wild bird.

**Group**	**ORFs**	**σC (amino aicd)**							**σNS (amino acid)**									
**(Isolates)**	**Position**	**24**	**71**	**106**	**113**	**134**	**135**		**72**	**121**	**152**	**158**	**160**	**162**	**218**	**254**	**273**	**350**
Commercial vaccine	S1133	T	T	T	T	D	V		D	P	R	A	S	P	A	L	T	M
	A15-19	T	T	T	T	D	V		**G**	**S**	**H**	**T**	**D**	**A**	**S**	**T**	**V**	**T**
	A15-48	T	T	T	T	D	V		**G**	**S**	**H**	**T**	**D**	**A**	**S**	**T**	**V**	**T**
	A15-71	T	T	T	T	D	V		**G**	**S**	**H**	**T**	**D**	**A**	**S**	**T**	**V**	**T**
	A15-108	T	T	T	T	D	V		**G**	**S**	**H**	**T**	**D**	**A**	**S**	**T**	**V**	**T**
	A15-113	T	T	T	T	D	V		**G**	**S**	**H**	**T**	**D**	**A**	**S**	**T**	**V**	**T**
Present study	A15-157	T	T	T	T	D	V		**G**	**S**	**H**	**T**	**D**	**A**	**S**	**T**	**V**	**T**
	A18-13	**N**	**T**	**R**	**I**	**D**	**I**		**G**	**S**	**H**	**T**	**D**	**A**	**S**	**T**	**V**	**T**
	A18-19	T	T	T	T	D	V		**G**	**S**	**H**	**T**	**D**	**A**	**S**	**T**	**V**	**T**
	A18-205	T	T	T	T	D	V		**G**	**S**	**H**	**T**	**D**	**A**	**S**	**T**	**V**	**T**
	A19-106	T	T	T	T	D	V		**G**	**S**	**H**	**T**	**D**	**A**	**S**	**T**	**V**	**T**
Attenuated vaccine	av-S1133	**N**	**T**	**R**	**I**	**D**	**I**		D	P	R	A	S	P	A	L	T	M
	SD09-1	N	T	R	I	D	I		D	P	R	A	S	P	A	L	T	M
	LN09-1	N	T	R	I	D	I		D	P	R	A	S	P	A	L	T	M
China	GX110116	N	T	R	I	D	I		D	P	R	A	S	P	A	L	T	M
	GuangxiR1	T	I	T	T	D	V		D	P	R	A	S	P	A	L	T	M
	GuangxiR2	T	I	T	T	D	V		D	P	R	A	S	P	A	L	T	M
California	K1600657	T	A	R	S	D	V		**G**	**S**	**H**	**T**	**D**	**A**	**S**	**T**	**V**	**T**
	SNU0044	-	A	T	M	D	V		-	-	-	-	-	-	-	-	-	-
	SNU0046	-	A	T	M	D	V		-	-	-	-	-	-	-	-	-	-
Korea	iReo0309	-	S	N	S	D	V		-	-	-	-	-	-	-	-	-	-
	ADL112770-ARV	-	-	-	-	-	-		D	S	H	T	N	A	S	T	V	-
	ADL112782-ARV	-	-	-	-	-	-		D	S	H	T	N	A	S	T	V	-
	ADL121187-ARV	-	-	-	-	-	-		D	S	H	T	N	A	S	T	V	-
	K738-14	T	S	M	L	S	V		D	S	H	T	N	A	S	T	V	T

*Deduced amino acid numbering corresponds to that of commercial vaccine isolate S1133, -: data is not available, specific deduced amino acid substitutions are showed in a bold*.

### Phylogenetic Analysis

**Figure 2:**
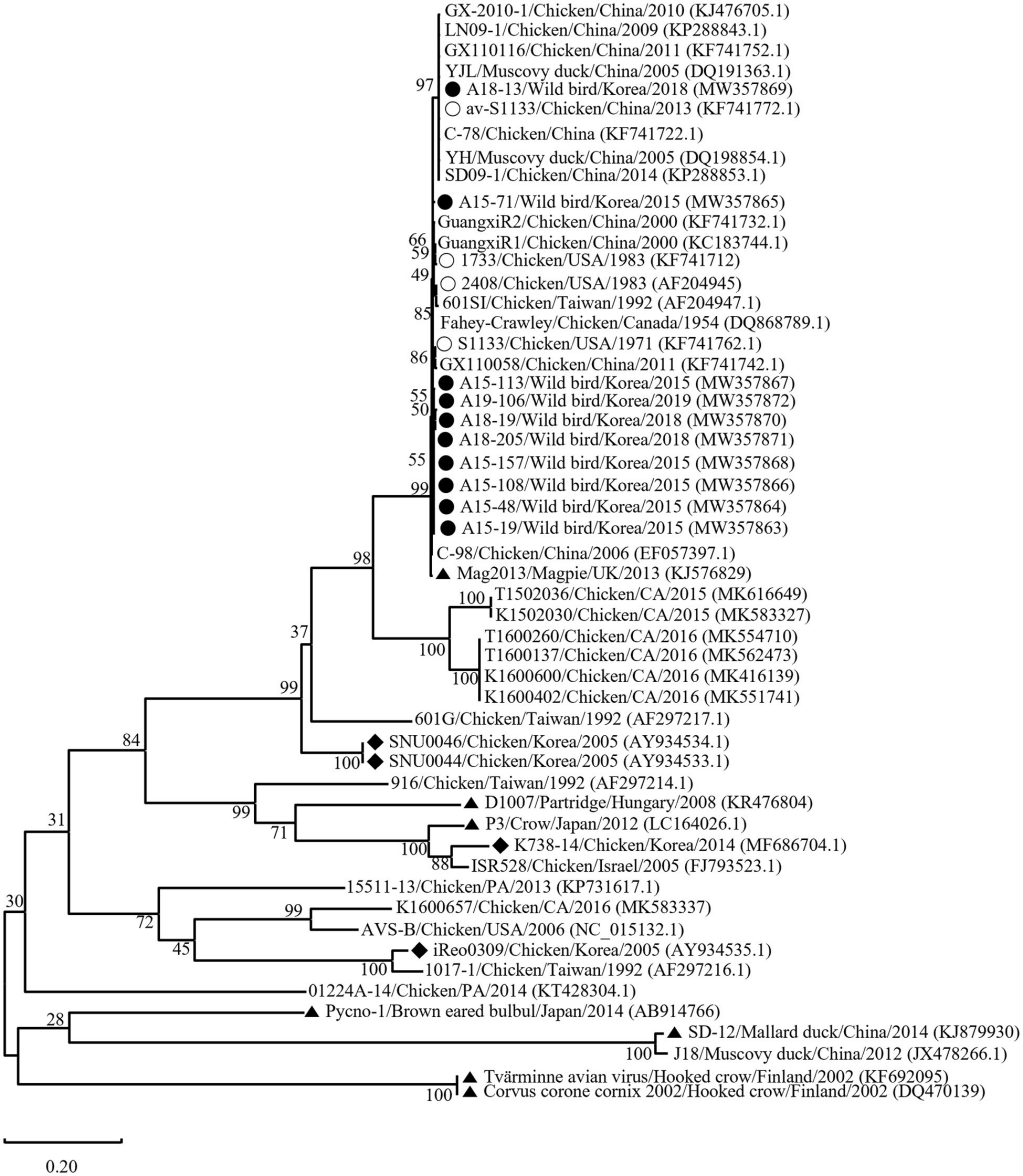
Maximum likelihood phylogenetic tree for the σC-encoding gene based on nucleotide sequences (981 nt). Maximum likelihood phylogenetic analyses were conducted using MEGA-X software with the Kimura 2-parameter model and 1,000 bootstrap replicates. The 43 reference sequences were obtained from GenBank. The black circle (•) indicates our isolates, while the white circle (°) indicates the vaccine isolates. Additionally, the black diamond (♦) indicates field isolates in Korea, and the black triangle (▴) indicates previously isolated wild bird isolates. Each sequence on the tree is identified by the isolate name, host, country of origin, year of isolation, and GenBank accession number.

**Figure 3:**
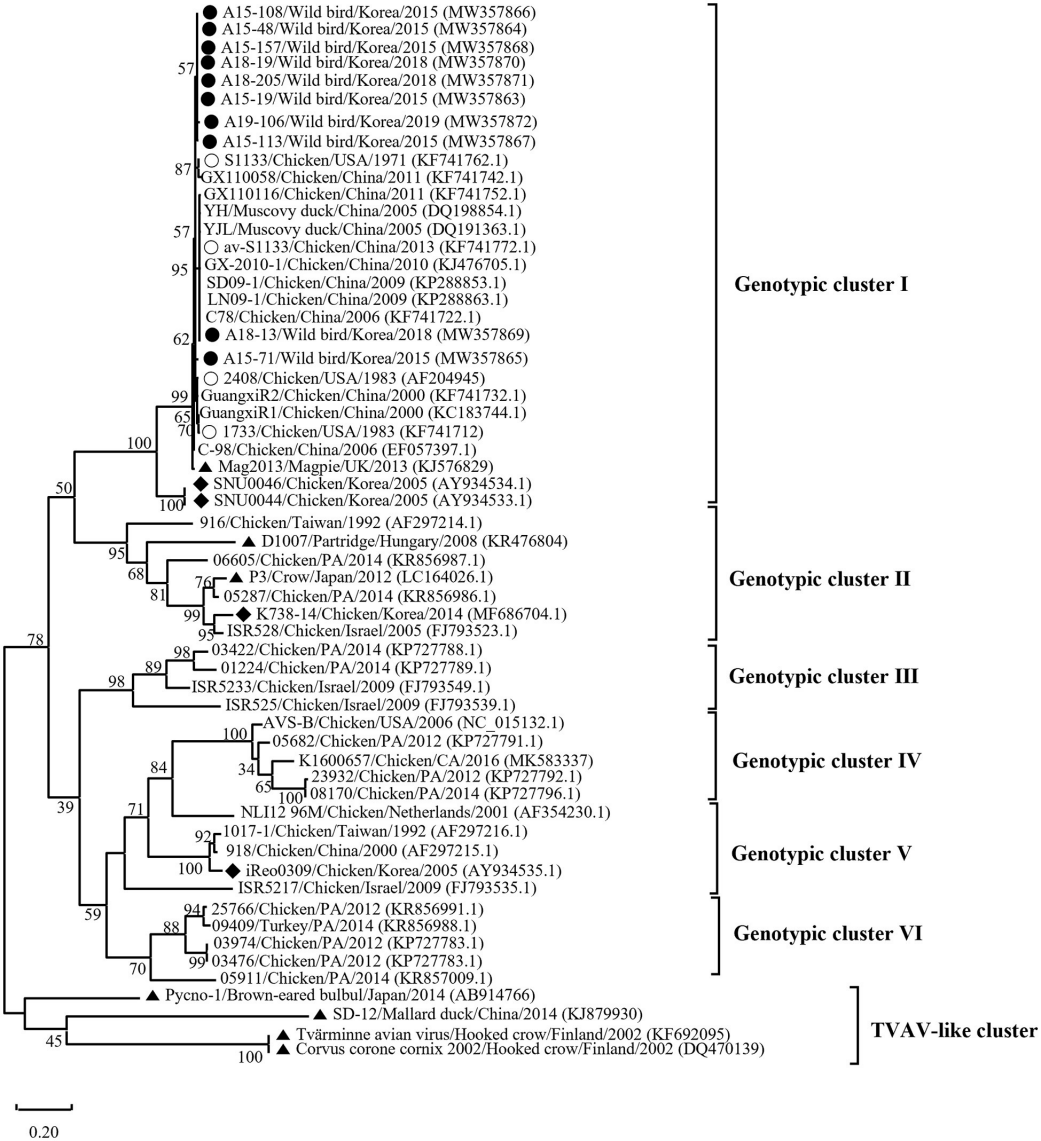
Phylogenetic tree of ARV isolates based on the deduced amino acid sequences of the σC-encoding gene. Maximum likelihood phylogenetic analyses were conducted using MEGA-X software with the Jones–Taylor–Thornton (JTT) model and 1,000 bootstrap replicates. The tree shows genetic relationship between the σC protein sequences (326 amino acids) of our 10 isolates and 48 reference isolates that were isolated from around the world. The virus isolates are clustered into six genotypic clusters. The black circle (•) indicates our isolates, and the white circle (°) indicates the vaccine isolates. Additionally, the black diamond (♦) indicates field isolates in Korea, and the black triangle (▴) indicates previously isolated wild bird isolates. Each sequence on the tree is identified by the isolate name, host, country of origin, year of isolation, and GenBank accession number.

**Figure 4:**
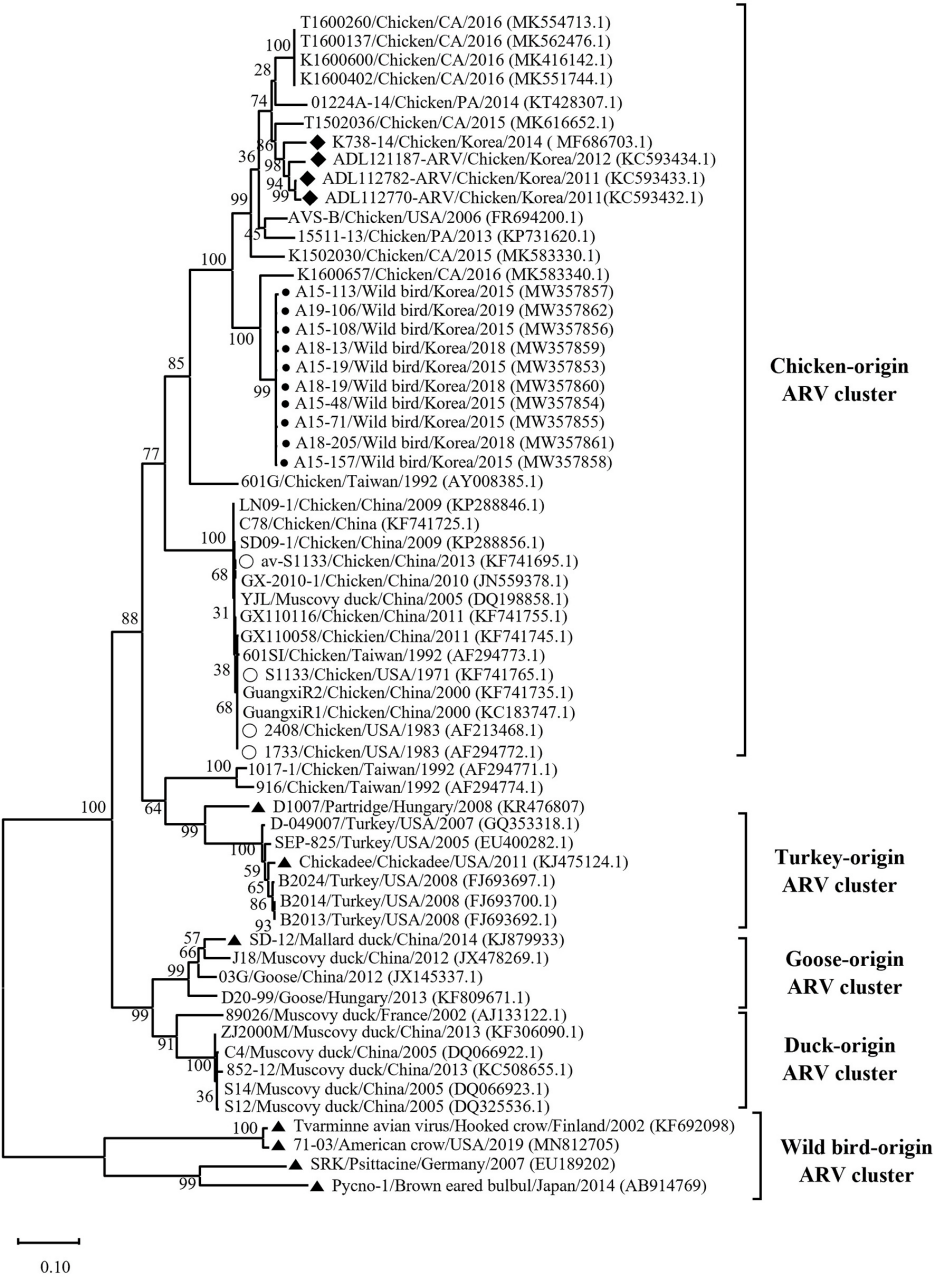
Maximum likelihood phylogenetic tree for the σNS-encoding gene based on nucleotide sequences (1,104 nt). Maximum likelihood phylogenetic analyses were conducted using MEGA-X software with the Kimura 2-parameter model and 1,000 bootstrap replicates. The 52 reference sequences were obtained from GenBank. The black circle (•) indicates our isolates, and the white circle (°) indicates the vaccine isolates. Additionally, the black diamond (♦) indicates field isolates in Korea, and the black triangle (▴) indicates previously isolated wild bird isolates. Each sequence on the tree is identified by the isolate name, host, country of origin, year of isolation, and GenBank accession number.

Phylogenetic analysis of the σC- and σNS-encoding genes was demonstrated. For the σC-encoding gene, nucleotide sequences of the 10 isolates showed that they were closely related to each other and clustered with Chinese chicken-origin isolates and commercial vaccine isolates. Conversely, previously identified TVAV-like wild bird isolates (Pycno-1, SD-12, Tvärminne avian virus, and Corvus corone cornix 2002) were distinct from all isolates in this study (Figure 2). To classify the genotypes of our isolates, we constructed phylogenetic trees using deduced amino acid sequences of the σC-encoding gene. This analysis revealed that all isolates were in the same cluster (genotypic cluster I) as the commercial vaccine isolates (Figure 3). For the σNS-encoding gene, nucleotide sequences of the 10 isolates showed that they are grouped together in one branch and clustered with the Californian chicken-origin isolate K1600657. Conversely, Chinese chicken-origin isolates, TVAV-like wild bird isolates (Pycno-1, SD-12, Tvärminne avian virus, 71-03, Chickadee, and SRK) and commercial vaccine isolates were distinct from all isolates in this study (Figure 4). For the p10- and p17-encoding gene, nucleotide sequences of the 10 isolates showed that they are clustered with Chinese chicken-origin isolates and commercial vaccine isolates. Conversely, previously identified TVAV-like wild bird isolates (Pycno-1 and Tvärminne avian virus) were distinct from all isolates in this study (Supplementary Figures 1, 2).

**Figure 5:**
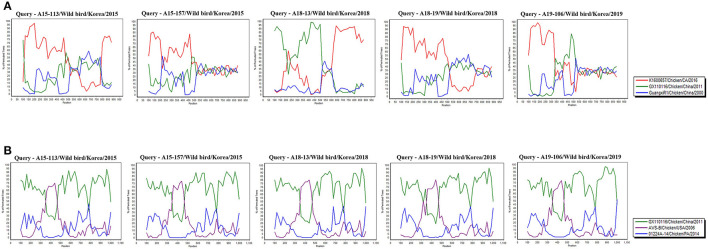
Bootscan analysis of wild bird ARV for detecting recombination was performed using Simplot program version 3.5.1, the neighbor-joining method with a Kimura 2-parameter applied and 100 replicates. **(A)** σC-encoding gene, **(B)** σNS-encoding gene.

In Recombination analysis, representative 5 isolates (A15-113, A15-157, A18-13, A18-19, and A19-106) were selected by five independent recombination patterns in the σC-encoding gene of our 10 isolates. A15-113 has a similar pattern to A15-108, and A15-157 has a similar pattern to A15-19, A15-48, and A18-205. In addition, A18-13 has a similar pattern to A15-71, and A18-19 and A19-106 showed independent patterns. Whereas all isolates have identical recombination patterns in σNS-encoding gene. All reference sequences in Supplementary Table 1 were analyzed. However, for the σC-encoding gene, meaningful recombination with 3 isolates (GX110116, GuangxiR1, and K1600657) were detected. And for the σNS-encoding gene, meaningful recombination with 3 isolates (GX110116, AVS-B, and 01224A-14) were detected. According to a Bootscan analysis, it appears that the σC-encoding gene of A18-13 were recombined with Chinese vaccine break isolates GX110116 and Californian chicken-origin isolate K1600657. However, 4 isolates (A15-113, A15-157, A18-19, and A19-106) did not show clear evidence for recombination with these isolates (GX110116 and K1600657). For the σNS-encoding gene, there is evidence that 5 isolates may have recombined with Chinese vaccine break isolate GX110116 and American chicken-origin isolate AVS-B (Figure 5).

## Discussion

It has been hypothesized that wild birds have a role in the transmission and maintenance of ARVs and pathogenic potential of ARVs in wild birds that usually commingle around poultry farms. However, lack of evidence to support these hypotheses. Molecular surveillance is crucial to strategize control and prevention of endemic diseases. Contrary to the previous study results, recently detected reovirus isolates in ostrich (*Stuthio camelus*) and free-living magpie were genetically related to chicken-origin ARVs (13, 31). Despite of the need for a reliable and relevant source of genetic information of wild birds, available genetic information is rare. In this study, we conducted surveillance and molecular characterization of ARVs in wild birds, and ARVs were detected in 1.9% (14/739) of the wild bird feces. This result suggests a much lower prevalence of ARVs than reported previously in some studies in Poland (11.5%; 9/78 dead wild birds; intestine) (12) and the USA (25.0%; 4/20 dead American woodcock; intestine) (49). Both studies reported that ARVs in wild bird's intestine had high prevalence than ARVs in wild bird feces. These high prevalence in intestines are probably due to ARVs have enteric tropism (50) and low prevalence in feces may have influenced by several factors, such as environmental factors or feces can act as an anti-viral factor (51). A similar result to our study was reported in Brazil that conducted surveillance of ARVs in fecal samples from broiler chickens (1.9%; 7/378) (52). The differences in prevalence could be attributed to the sample type (feces *vs*. tissue) or regional differences.

We classified reovirus isolates in this study based on the σC protein, all isolates belonged to genotypic cluster I, which is the predominant cluster in chicken-origin ARVs and includes commercial vaccine isolates (Figure 3) (8, 53). Similar results in wild birds were described in the UK and ARV isolated from free-living magpie (*Pica pica*) belonged to genotypic cluster I (31). Conversely, in previous studies, wild bird-origin ARVs belonged to the TVAV-like cluster (27–30, 34, 35). Moreover, the phylogenetic analysis of the p10- and p17-encoding genes revealed that all isolates were closely clustered with Chinese chicken-origin isolates. For the σNS-encoding gene, our isolates were more closely clustered with the Californian chicken-origin isolate K1600657 that belonged to chicken-origin ARV cluster (Figure 4). These findings suggest that chicken-origin ARV can infect wild birds and can thus be a potential source of ARV infections that can spillover to chickens and vice versa.

Throughout this study, the 10 ARVs of the σC protein showed high degree of antigenic homogeneity to commercial vaccine isolates (inactivated vaccine) and Chinese chicken-origin isolates. However, these isolates were distinct from Korean chicken-origin isolates. In addition, analysis of the σNS protein showed high similarity to Californian chicken-origin isolate K1600657. Our results indicated that the deduced amino acid substitution patterns of all isolates are identical to those of chicken-origin ARVs in the σC and σNS proteins. Particularly, the A18-13 isolate had identical patterns with avirulent ARV (av-S1133) and virulent ARV (GX110116) in terms of the σC protein, which contributed to ARV virulence (54, 55). For the σNS protein, deduced amino acid substitutions at positions 72, 121, 152, 158, 160, 162, 218, 254, 273, and 350 were observed for all isolates with respect to S1133 (Table 3). These substitutions were also observed for the Californian chicken-origin isolate K1600657 that was associated with severe clinical signs (53). These findings suggested that pathotyping based on the σC protein has limitations. More analyses of polymorphisms and deduced amino acid substitutions of other segments are required for pathotyping of ARVs. For the p10 protein, all isolates had a putative transmembrane domains, which are members of the fusion associated small transmembrane (FAST) protein family that conserved in chicken-origin ARVS (56). For the p17 protein, all isolates had identical nuclear localization signal (NLS) motif with chicken-origin isolates, which is conserved in chicken-origin ARVS (Supplementary Table 5) (57). These finding suggested that 10 isolates have identical FAST protein and NLS to chicken-origin ARVs.

According to recombination analysis, the σC-encoding gene of A18-13 isolates might have recombined with Chinese vaccine break isolate GX110116 at ~300–500 nucleotide sequence positions that include four non-synonymous nucleotide substitutions (G^317^, T^338^, A^403^, and C^405^) and Californian chicken-origin isolate K1600657 at approximate 700–900 nucleotide sequence positions with no non-synonymous nucleotide substitutions. Whereas other four isolates showed independent patterns in recombination analysis, although these isolates did not show clear evidence of recombination. The σNS-encoding gene of five isolates (A15-113, A15-157, A18-13, A18-19, and A19-106) might have recombined with American chicken-origin isolate AVS-B at ~350–470 nucleotide sequence positions that include three non-synonymous nucleotide substitutions (T^361^, A^455^, and A^472^) and Chinese vaccine break isolate GX110116 at 100–350 and 470–1,000 nucleotide sequence positions that include seven non-synonymous nucleotide substitutions (G^215^, G^478^, G^484^, T^652^, A^760^, G^817^, and C^1049^). In addition, all isolates showed identical recombination patterns in the σNS-encoding gene (Figure 5 and Supplementary Table 4). These findings suggest that recombination patterns are seems to be associated with the characteristics of the σC and σNS protein. In terms of the σC protein, which is the most variable region in the viral genome (58). For the σNS protein, which is relatively conserved among other genome segments (59). Moreover, our 10 isolates genome reflects genomic reassortment events and intra-segmental recombination between Chinese vaccine break isolate and American chicken-origin isolate in the σNS-encoding gene (Figure 5). These finding indicated that our isolates are intracontinental recombined ARVs and can be potential risk in commercial chicken farms.

Wild bird ARVs have been isolated in sedentary bird species, including magpie, crow, partridge, black-capped chickadee, and psittacine birds (27, 29, 31–33). A previous study reported the isolation of wild bird ARV from a migratory bird species (mallard duck) (35). Eight isolates identified in this study (A15-48, A15-71, A15-108, A15-113, A18-13, A18-19, A18-205, and A19-106) were isolated from migratory bird species (bean goose, Eurasian teal, Indian spot-billed duck, and mallard duck) (Table 2). In the flyways of these birds, South Korea is an important wintering site for wild migratory birds, such as waterfowl which flies across the East Asia–Australian flyway, with Korea and China located along this flyway (60). The A15-157 isolate was from an oriental turtle dove. Notably, the oriental turtle dove is a sedentary bird species commonly found around chicken farms in Korea. These sedentary bird species may play a role in ARV transmission between poultry and wild birds.

## Conclusion

We describe genetic characterization of 10 ARVs isolated from wild birds' feces and showed that all isolates were closely related to chicken-origin ARVs. It is possible that wild birds may play a potential role in the epidemiology of chicken-origin ARVs as a carrier. For further study to evaluate risk factors, it is essential to conduct whole-genome sequencing and investigate the potential pathogenicity of ARVs isolated from wild birds for domestic poultry.

## Data Availability Statement

The datasets presented in this study can be found in online repositories. The names of the repository/repositories and accession number(s) can be found in the article/Supplementary Material.

## Author Contributions

S-YC and MK: contributed to conception and design of experiments. H-KJ, S-WK, Y-RC, KS, BW, J-YP, and J-FZ: contributed to acquisition, analysis, and interpretation of data. S-WK, Y-RC, S-YC, and MK: drafted and/or revised the article. All authors have read and agreed to the published version of the manuscript.

## Funding

This work was supported by Korea Institute of Planning and Evaluation for Technology in Food, Agriculture and Forestry (IPET) through Agriculture, Food and Rural Affairs Convergence Technologies Program for Educating Creative Global Leader (716002-7, 320005-4) funded by Ministry of Agriculture, Food and Rural Affairs (MAFRA). The funders had no role in study design, data collection and analysis, decision to publish, or preparation of the manuscript.

## Conflict of Interest

The authors declare that the research was conducted in the absence of any commercial or financial relationships that could be construed as a potential conflict of interest.

## Publisher's Note

All claims expressed in this article are solely those of the authors and do not necessarily represent those of their affiliated organizations, or those of the publisher, the editors and the reviewers. Any product that may be evaluated in this article, or claim that may be made by its manufacturer, is not guaranteed or endorsed by the publisher.

## Supplementary Material

Supplementary Figure 1 Maximum likely phylogenetic tree for p10-encoding gene based on nucleotide sequences (297 nt). Maximum likelihood phylogenetic analyses were conducted using MEGA X software with the Kimura 2-parameter model and 1,000 bootstrap replicates. The 31 reference sequences were obtained from GenBank. The black circle (•) indicates our isolates while the white circle (°) indicates the vaccine isolates. Additionally, the black diamond (♦) indicates field isolates in Korea while the black triangle (▴) indicates previously isolated wild bird isolates. Each sequence on the tree is identified by isolated name, host and country of origin, year of isolation, GenBank accession number.

Supplementary Figure 2 Maximum likely phylogenetic tree for p17-encoding gene based on nucleotide sequences (441 nt). Maximum likelihood phylogenetic analyses were conducted using MEGA X software with the Kimura 2-parameter model and 1,000 bootstrap replicates. The 31 reference sequences were obtained from GenBank. The black circle (•) indicates our isolates while the white circle (°) indicates the vaccine isolates. Additionally, the black diamond (♦) indicates field isolates in Korea while the black triangle (▴) indicates previously isolated wild bird isolates. Each sequence on the tree is identified by isolated name, host and country of origin, year of isolation, GenBank accession number.

Supplementary Table 1 Information of new isolated ARV and reference isolates used in this study.

Supplementary Table 2 Sequence alignment of the σC-encoding gene (981 nt).

Supplementary Table 3 Sequence alignment of the σNS-encoding gene (1,104 nt).

Supplementary Table 4 Single nucleotide polymorphisms in the σC- and σNS-encoding genes of ARV isolates from wild bird.

Supplementary Table 5 Deduced amino acid substitutions in the putative transmembrane domains of the p10 and nuclear localization signal motif in the p17.
