# Supplementary material for: Isolation and Genomic Characterization of Avian Reovirus From Wild Birds in South Korea
Source: Front Vet Sci. 2022 Jan 28;9:794934. doi: 10.3389/fvets.2022.794934 (PMC8831841; doi:10.3389/fvets.2022.794934)
Supplement: Supplementary Figure 1 — Maximum likely phylogenetic tree for p10-encoding gene based on nucleotide sequences (297 nt). Maximum likelihood phylogenetic analyses were conducted using MEGA X software with the Kimura 2-parameter model and 1,000 bootstrap replicates. The 31 reference sequences were obtained from GenBank. The black circle (•) indicates our isolates while the white circle (°) indicates the vaccine isolates. Additionally, the black diamond (♦) indicates field isolates in Korea while the black triangle (▴) indicates previously isolated wild bird isolates. Each sequence on the tree is identified by isolated name, host and country of origin, year of isolation, GenBank accession number. [file Table_1.DOCX]

Supplementary Material

# Supplementary Figures

## Supplementary Figure 1

Maximum likely phylogenetic tree for the p10-encoding gene based on nucleotide sequences (297 nt)

71

32

45

71

32

33

96

97

83

99

91

37

41

59

89

● A15-71/Wild bird/Korea/2015 (MW357865)

○ 2408/Chicken/USA/1983 (AY436605.1)

● A15-108/Wild bird/Korea/2015 (MW357866)

● A15-113/Wild bird/Korea/2015 (MW357867)

● A18-19/Wild bird/Korea/2018 (MW357870)

● A18-205/Wild bird/Korea/2018 (MW357871)

● A19-106/Wild bird/Korea/2019 (MW357872)

C-98/Chicken/China/2006 (EF057397.1)

● A15-48/Wild bird/Korea/2015 (MW357864)

Fahey-Crawley/Chicken/Canada/1954 (DQ868789.1)

GuangxiR2/Chicken/China/2000 (KF741732.1)

GX110058/Chicken/China/2011 (KF741742.1)

○S1133/Chicken/USA/1971 (KF741762.1)

● A15-19/Wild bird/Korea/2015 (MW357863)

● A15-157/Wild bird/Korea/2015 (MW357868)

○1733/Chicken/USA/1983 (KF741712)

○ av-S1133/Chicken/China/2013 (KF741772.1)

GX-2010-1/Chicken/China/2010 (KJ476705.1)

● A18-13/Wild bird/Korea/2018 (MW357869)

GX110116/Chicken/China/2011 (KF741752.1)

C78/Chicken/China (KF741722.1)

YJL/Muscovy duck/China/2005 (DQ191363.1)

YH/Muscovy duck/China/2005 (DQ198854.1)

SD09-1/Chicken/China/2014 (KP288853.1)

LN09-1/Chicken/China/2009 (KP288843.1)

GuangxiR1/Chicken/China/2000 (KC183744.1)

916/Chicken/Taiwan/1992 (AY436604.1)

T1502036/Chicken/CA/2015 (MK616649.1)

K1502030/Chicken/CA/2015 (MK583327.1)

T1600260/Chicken/CA/2016 (MK554710)

T1600137/Chicken/CA/2016 (MK562473.1)

K1600600/Chicken/CA/2016 (MK416139.1)

K1600657/Chicken/CA/2016 (MK583337.1)

AVS-B/Chicken/USA/2006 (NC_015132.1)

01224A-14/Chicken/PA/2014 (KT428304.1)

▲ P3/Crow/Japan/2012 (LC164026.1)

▲ D1007/Partridge/Hungary/2008 (KR476804.1)

15511-13/Chicken/PA/2013 (KP731617.1)

▲ Tvarminne avian reovirus/Hooked crow/Finland/2002 (KF692095.1)

▲ Pycno-1/Brown eared bulbul/Japan/2014 (AB914766.1)

J18/Muscovy duck/China/2012 (JX478266.1)

0.10

## Supplementary Figure 2

## Maximum likely phylogenetic tree for the p17-encoding gene based on nucleotide sequences (441 nt)

○ 2408/Chicken/USA/1983 (AY438594.1)

○ 1733/Chicken/USA/1983 (KF741712)

● A15-19/Wild bird/Korea/2015 (MW357863)

● A15-108/Wild bird/Korea/2015 (MW357866)

● A15-157/Wild bird/Korea/2015 (MW357868)

● A18-13/Wild bird/Korea/2018 (MW357869)

C-98/Chicken/China/2006 (EF057397.1)

Fahey-Crawley/Chicken/Canada/1954 (DQ868789.1)

GX-2010-1/Chicken/China/2010 (KJ476705.1)

GX110058/Chicken/China/2011 (KF741742.1)

YJL/Muscovy duck/China/2005 (DQ191363.1)

YH/Muscovy duck/China/2005 (DQ198854.1)

○ av-S1133/Chicken/China/2013 (KF741772.1)

SD09-1/Chicken/China/2014 (KP288853.1)

LN09-1/Chicken/China/2009 (KP288843.1)

GX110116/Chicken/China/2011 (KF741752.1)

● A15-113/Wild bird/Korea/2015 (MW357867)

● A19-106/Wild bird/Korea/2019 (MW357872)

● A15-48/Wild bird/Korea/2015 (MW357864)

● A18-205/Wild bird/Korea/2018 (MW357871)

● A15-71/Wild bird/Korea/2015 (MW357865)

GuangxiR2/Chicken/China/2000 (KF741732.1)

○ S1133/Chicken/USA/1971 (KF741762.1)

C78/Chicken/China (KF741722.1)

● A18-19/Wild bird/Korea/2018 (MW357870)

GuangxiR1/Chicken/China/2000 (KC183744.1)

916/Chicken/Taiwan/1992 (AY438593.1)

T1502036/Chicken/CA/2015 (MK616649.1)

K1502030/Chicken/CA/2015 (MK583327.1)

T1600260/Chicken/CA/2016 (MK554710)

T1600137/Chicken/CA/2016 (MK562473.1)

K1600600/Chicken/CA/2016 (MK416139.1)

▲ Pycno-1/Brown eared bulbul/Japan/2014 (AB914766.1)

▲ P3/Crow/Japan/2012 (LC164026.1)

▲ D1007/Partridge/Hungary/2008 (KR476804.1)

01224A-14/Chicken/PA/2014 (KT428304.1)

15511-13/Chicken/PA/2013 (KP731617.1)

K1600657/Chicken/CA/2016 (MK583337.1)

AVS-B/Chicken/USA/2006 (NC_015132.1)

▲ Tvarminne avian reovirus/Hooked crow/Finland/2002 (KF692095.1)

J18/Muscovy duck/China/2012 (JX478266.1)

0.20

97

61

84

55

84

99

95

77

74

99

95

100

87

38

# Supplementary Tables

## Supplementary Table 1

Information of new isolated ARV and reference isolates used in this study

| Number | Group  (isolates) | Isolate | Host | Origin | year | GenBank accession  number | |
| --- | --- | --- | --- | --- | --- | --- | --- |
|  |  |  |  |  |  | σC | σNS |
| 1 | Korea | SNU0044 | Chicken | Korea | 2005 | AY934533.1 | NA^a^ |
| 2 |  | SNU0046 | Chicken | Korea | 2005 | AY934534.1 | NA |
| 3 |  | K738-14 | Chicken | Korea | 2014 | MF686704.1 | MF686703.1 |
| 4 |  | iReo0309 | Chicken | Korea | 2005 | AY934535.1 | NA |
| 5 |  | ADL112770-ARV | Chicken | Korea | 2011 | NA | KC593432.1 |
| 6 |  | ADL112782-ARV | Chicken | Korea | 2011 | NA | KC593433.1 |
| 7 |  | ADL121187-ARV | Chicken | Korea | 2012 | NA | KC593434.1 |
| 8 | China | 601G | Chicken | Taiwan | 1992 | AF297217.1 | AY008385.1 |
| 9 |  | 601SI | Chicken | Taiwan | 1992 | AF204947.1 | AF294773.1 |
| 10 |  | 916 | Chicken | Taiwan | 1992 | AF297214.1 | AF294774.1 |
| 11 |  | 1017-1 | Chicken | Taiwan | 1992 | AF297216.1 | AF294771.1 |
| 12 |  | GuangxiR1 | Chicken | China | 2000 | KC183744.1 | KC183747.1 |
| 13 |  | GuangxiR2 | Chicken | China | 2000 | KF741732.1 | KF741735.1 |
| 14 |  | C78 | Chicken | China | 2006 | KF741722.1 | KF741725.1 |
| 15 |  | C-98 | Chicken | China | 2006 | EF057397.1 | NA |
| 16 |  | GX-2010-1 | Chicken | China | 2010 | KJ476705.1 | JN559378.1 |
| 17 |  | GX110058 | Chicken | China | 2011 | KF741742.1 | KF741745.1 |
| 18 |  | 03G | Goose | China | 2012 | KF729962.1 | JX145337.1 |
| 19 |  | C4 | Muscovy duck | China | 2005 | DQ066924.1 | DQ066922.1 |
| 20 |  | S12 | Muscovy duck | China | 2005 | DQ643970.1 | DQ325536.1 |
| 21 |  | S14 | Muscovy duck | China | 2005 | DQ066925.1 | DQ066923.1 |
| 22 |  | YH | Muscovy duck | China | 2005 | DQ198854.1 | NA |
| 23 |  | YJL | Muscovy duck | China | 2005 | DQ191363.1 | DQ198858.1 |
| 24 |  | J18 | Muscovy duck | China | 2012 | JX478266.1 | JX478269.1 |
| 25 |  | 852-12 | Muscovy duck | China | 2013 | KC508656.1 | KC508655.1 |
| 26 |  | ZJ2000M | Muscovy duck | China | 2013 | KF306091.1 | KF306090.1 |
| 27 | Vaccine break | LN09-1 | Chicken | China | 2009 | KP288843.1 | KP288846.1 |
| 28 |  | SD09-1 | Chicken | China | 2009 | KP288853.1 | KP288856.1 |
| 29 |  | GX110116 | Chicken | China | 2011 | KF741752.1 | KF741755.1 |
| 30 | Israel | ISR528 | Chicken | Israel | 2005 | FJ793523.1 | NA |
| 31 | France | 89026 | Muscovy duck | France | 2002 | AJ310525.1 | AJ133122.1 |
| 32 | Hungary | D20-99 | Goose | Hungary | 2013 | KF809668.1 | KF809671.1 |
| 33 | Canada | Fahey-Crawley | Chicken | Canada | 1954 | DQ868789.1 | NA |
| 34 | Attenuated vaccine | av-S1133 | Chicken | China | 2013 | KF741772.1 | KF741695.1 |
| 35 | Commercial vaccine | S1133 | Chicken | USA | 1971 | KF741762.1 | KF741765.1 |
| 36 |  | 1733 | Chicken | USA | 1983 | KF741712 | AF294772.1 |
| 37 |  | 2408 | Chicken | USA | 1983 | AF204945 | AF213468.1 |
| 38 | USA | AVS-B | Chicken | USA | 2006 | NC_015132.1 | FR694200.1 |
| 39 |  | 15511-13 | Chicken | PA | 2013 | KP731617.1 | KP731620.1 |
| 40 |  | 01224A-14 | Chicken | PA | 2014 | KT428304.1 | KT428307.1 |
| 41 |  | K1502030 | Chicken | CA | 2015 | MK583327 | MK583330.1 |
| 42 |  | T1502036 | Chicken | CA | 2015 | MK616649 | MK616652.1 |
| 43 |  | K1600402 | Chicken | CA | 2016 | MK551741 | MK551744.1 |
| 44 |  | K1600600 | Chicken | CA | 2016 | MK416139 | MK416142.1 |
| 45 |  | K1600657 | Chicken | CA | 2016 | MK583337 | MK583340.1 |
| 46 |  | T1600137 | Chicken | CA | 2016 | MK562473 | MK562476.1 |
| 47 |  | T1600260 | Chicken | CA | 2016 | MK554710 | MK554713.1 |
| 48 |  | SEP-825 | Turkey | USA | 2005 | NA | EU400282.1 |
| 49 |  | D-049007 | Turkey | USA | 2007 | NA | GQ353318.1 |
| 50 |  | B2013 | Turkey | USA | 2008 | NA | FJ693692.1 |
| 51 |  | B2014 | Turkey | USA | 2008 | NA | FJ693700.1 |
| 52 |  | B2024 | Turkey | USA | 2008 | NA | FJ693697.1 |
| 53 | Chicken-origin ARV  like Wild bird | P3 | Crow | Japan | 2012 | LC164026.1 | NA |
| 54 |  | Mag2013 | Magpie | UK | 2013 | KJ576829 | NA |
| 55 |  | D1007 | Partridge | Hungary | 2008 | KR476804 | KR476807 |
| 56 | Tvärminne avian virus  (TVAV)-like wild bird | SD-12 | Mallard duck | China | 2014 | KJ879930 | KJ879933 |
| 57 |  | Pycno-1 | Brown eared bulbul | Japan | 2014 | AB914766 | AB914769 |
| 58 |  | 71-03 | American crow | USA | 2019 | NA | MN812705 |
| 59 |  | Corvus corone cornix2002 | Hooked crow | Finland | 2002 | DQ470139 | NA |
| 60 |  | Tvärminne avian virus | Hooked crow | Finland | 2002 | KF692095 | KF692098 |
| 61 |  | Chickadee | Chickadee | USA | 2011 | NA | KJ475124.1 |
| 62 |  | SRK | Psittacine | Germany | 2007 | NA | EU189202 |

^a^ NA, Nucleotide sequences are not available.

## Supplementary Table 2

Sequence alignment of the σC-encoding gene (981 nt)

| No. | Isolate | av-S1133 | S1133 | 2408 | 1733 | GuangxiR1 | SD09-1 | LN09-1 | GX110116 | | K1600657 | SNU0044 | SNU0046 | K738-14 | iREO0309 | SD-12 | Pycno-1 | Corvus corone comix 2002 | Tvärminne avian virus |
| --- | --- | --- | --- | --- | --- | --- | --- | --- | --- | --- | --- | --- | --- | --- | --- | --- | --- | --- | --- |
| 1 | A15-19 | 98.88 | 99.08 | 99.08 | 99.29 | 99.49 | 99.06 | 98.98 | 98.88 | 55.09 | | 51.80 | 74.52 | 62.21 | 51.80 | 48.65 | 53.54 | 49.38 | 49.90 |
| 2 | A15-48 | 98.98 | 99.19 | 99.19 | 99.39 | 99.59 | 99.16 | 99.08 | 98.98 | 55.30 | | 51.80 | 74.52 | 62.51 | 51.80 | 48.65 | 53.76 | 49.48 | 50.00 |
| 3 | A15-71 | 98.68 | 99.29 | 98.88 | 99.08 | 99.29 | 98.85 | 98.78 | 98.68 | 54.79 | | 51.80 | 74.32 | 62.31 | 51.80 | 48.75 | 52.80 | 49.27 | 49.80 |
| 4 | A15-108 | 98.78 | 98.98 | 98.98 | 99.19 | 99.39 | 98.96 | 98.88 | 98.78 | 55.44 | | 51.80 | 74.52 | 62.51 | 51.80 | 49.10 | 53.94 | 49.38 | 49.90 |
| 5 | A15-113 | 98.68 | 98.88 | 98.88 | 99.08 | 99.29 | 98.85 | 98.78 | 98.68 | 55.19 | | 50.96 | 74.32 | 62.41 | 50.96 | 48.46 | 53.64 | 50.21 | 50.70 |
| 6 | A15-157 | 99.08 | 99.29 | 99.29 | 99.49 | 99.69 | 99.27 | 99.19 | 99.08 | 55.19 | | 51.80 | 74.52 | 62.41 | 51.80 | 48.65 | 53.64 | 49.38 | 49.90 |
| 7 | A18-13 | 99.39 | 98.37 | 98.37 | 98.57 | 98.78 | 99.58 | 99.49 | 99.39 | 54.79 | | 51.99 | 74.13 | 62.21 | 51.99 | 48.96 | 53.75 | 49.58 | 50.10 |
| 8 | A18-19 | 98.98 | 99.08 | 99.08 | 99.29 | 99.49 | 99.16 | 99.08 | 98.98 | 55.19 | | 51.80 | 74.32 | 62.41 | 51.80 | 48.75 | 53.64 | 49.48 | 50.00 |
| 9 | A18-205 | 99.08 | 99.29 | 99.29 | 99.49 | 99.69 | 99.27 | 99.19 | 99.08 | 55.19 | | 51.80 | 74.52 | 62.41 | 51.80 | 48.65 | 53.6 | 49.38 | 49.90 |
| 10 | A19-106 | 98.88 | 98.98 | 99.98 | 99.19 | 99.39 | 99.06 | 98.98 | 98.88 | 55.30 | | 50.29 | 74.32 | 62.11 | 50.30 | 48.46 | 53.64 | 49.27 | 49.80 |

## Supplementary Table 3

Sequence alignment of the σNS-encoding gene (1,104 nt)

| No. | Isolate | av-S1133 | S1133 | 2408 | 1733 | GuangxiR1 | SD09-1 | LN09-1 | GX110116 | K1600657 | K738-14 | ADL112770-ARV | ADL112782-ARV | ADL121187-ARV | SD-12 | Pycno-1 | 71-03 | Tvärminne avian virus | Chickadee | SRK |
| --- | --- | --- | --- | --- | --- | --- | --- | --- | --- | --- | --- | --- | --- | --- | --- | --- | --- | --- | --- | --- |
| 1 | A15-19 | 81.60 | 81.69 | 81.78 | 81.78 | 81.78 | 81.78 | 81.87 | 81.60 | 92.94 | 87.23 | 86.59 | 87.01 | 86.07 | 76.16 | 58.96 | 58.99 | 59.30 | 77.54 | 58.51 |
| 2 | A15-48 | 81.60 | 81.69 | 81.78 | 81.78 | 81.78 | 81.78 | 81.87 | 81.60 | 92.94 | 87.32 | 86.69 | 87.11 | 86.18 | 76.16 | 58.96 | 58.99 | 59.30 | 77.63 | 58.51 |
| 3 | A15-71 | 81.41 | 81.51 | 81.60 | 81.60 | 81.60 | 81.60 | 81.69 | 81.41 | 92.75 | 87.14 | 86.49 | 86.90 | 85.97 | 75.98 | 58.96 | 58.81 | 59.12 | 77.76 | 58.51 |
| 4 | A15-108 | 81.32 | 81.41 | 81.51 | 81.51 | 81.51 | 81.51 | 81.60 | 81.32 | 92.66 | 87.23 | 86.59 | 87.01 | 86.07 | 76.16 | 58.83 | 59.17 | 59.48 | 77.63 | 58.42 |
| 5 | A15-113 | 81.32 | 81.41 | 81.51 | 81.51 | 81.51 | 81.51 | 81.60 | 81.32 | 92.75 | 87.05 | 86.49 | 86.90 | 85.98 | 76.07 | 58.40 | 58.94 | 58.99 | 77.63 | 58.42 |
| 6 | A15-157 | 81.51 | 81.60 | 81.69 | 81.69 | 81.69 | 81.69 | 81.78 | 81.51 | 92.84 | 87.23 | 86.59 | 87.01 | 86.07 | 76.07 | 59.05 | 58.99 | 59.30 | 77.54 | 58.60 |
| 7 | A18-13 | 81.51 | 81.60 | 81.69 | 81.69 | 81.69 | 81.69 | 81.78 | 81.51 | 92.84 | 87.23 | 86.49 | 86.90 | 85.97 | 76.07 | 58.78 | 58.72 | 59.03 | 77.44 | 58.24 |
| 8 | A18-19 | 81.60 | 81.60 | 81.78 | 81.78 | 81.78 | 81.78 | 81.87 | 81.60 | 92.94 | 87.32 | 86.69 | 87.11 | 86.18 | 76.16 | 58.96 | 58.99 | 59.30 | 77.63 | 58.51 |
| 9 | A18-205 | 81.51 | 81.60 | 81.69 | 81.69 | 81.69 | 81.69 | 81.78 | 81.51 | 92.84 | 87.23 | 86.59 | 87.01 | 86.07 | 76.07 | 58.87 | 58.90 | 59.21 | 77.54 | 58.42 |
| 10 | A19-106 | 81.69 | 81.78 | 81.87 | 81.87 | 81.87 | 81.87 | 81.96 | 81.69 | 92.84 | 87.23 | 86.59 | 87.01 | 86.07 | 76.25 | 58.87 | 58.99 | 59.30 | 77.73 | 58.42 |

## Supplementary Table 4

Single nucleotide polymorphisms in the σC- and σNS-encoding genes of ARV isolates from wild bird

| Group  (isolates) | ORFs | | | σC (nucleotide) | | | |  | | | | | σNS (nucleotide) | | | | | | | |
| --- | --- | --- | --- | --- | --- | --- | --- | --- | --- | --- | --- | --- | --- | --- | --- | --- | --- | --- | --- | --- |
|  | Position | 71 | 213 | 317 | 338 | 403 | 405 |  | 215 | 361 | 455 | 472 | | 478 | 484 | 652 | 760 | 817 | 1049 |  |
| Attenuated vaccine | av-S1133 | **A** | **A** | **G** | **T** | **A** | **C** |  | A | C | G | G | | A | C | G | C | A | T |  |
| Commercial vaccine | S1133 | C | C | C | C | G | A |  | A | C | G | G | | A | C | G | C | A | T |  |
|  | 2408 | C | C | C | C | G | A |  | A | C | G | G | | A | C | G | C | A | T |  |
|  | 1733 | C | C | C | C | G | A |  | A | C | G | G | | A | C | G | C | A | T |  |
| China | SD09-1 | A | A | G | T | A | C |  | A | C | G | G | | A | C | G | C | A | T |  |
|  | LN09-1 | A | A | G | T | A | C |  | A | C | G | G | | A | C | G | C | A | T |  |
|  | GX110116 | A | A | G | T | A | C |  | A | C | G | G | | A | C | G | C | A | T |  |
|  | GuangxiR1 | C | C | C | C | G | A |  | A | C | G | G | | A | C | G | C | A | T |  |
|  | GuangxiR2 | C | C | C | C | G | A |  | A | C | G | G | | A | C | G | C | A | T |  |
| California | K1600657 | C | T | G | C | G | G |  | **G** | **T** | **A** | **A** | | **G** | **G** | **T** | **A** | **G** | **C** |  |
| Present study | A15-19 | C | C | C | C | G | A |  | **G** | **T** | **A** | **A** | | **G** | **G** | **T** | **A** | **G** | **C** |  |
|  | A15-48 | C | C | C | C | G | A |  | **G** | **T** | **A** | **A** | | **G** | **G** | **T** | **A** | **G** | **C** |  |
|  | A15-71 | C | C | C | C | G | A |  | **G** | **T** | **A** | **A** | | **G** | **G** | **T** | **A** | **G** | **C** |  |
|  | A15-108 | C | C | C | C | G | A |  | **G** | **T** | **A** | **A** | | **G** | **G** | **T** | **A** | **G** | **C** |  |
|  | A15-113 | C | C | C | C | G | A |  | **G** | **T** | **A** | **A** | | **G** | **G** | **T** | **A** | **G** | **C** |  |
|  | A15-157 | C | C | C | C | G | A |  | **G** | **T** | **A** | **A** | | **G** | **G** | **T** | **A** | **G** | **C** |  |
|  | A18-13 | **A** | **A** | **G** | **T** | **A** | **C** |  | **G** | **T** | **A** | **A** | | **G** | **G** | **T** | **A** | **G** | **C** |  |
|  | A18-19 | C | T | C | C | G | A |  | **G** | **T** | **A** | **A** | | **G** | **G** | **T** | **A** | **G** | **C** |  |
|  | A18-205 | C | C | C | C | G | A |  | **G** | **T** | **A** | **A** | | **G** | **G** | **T** | **A** | **G** | **C** |  |
|  | A19-106 | C | T | C | C | G | A |  | **G** | **T** | **A** | **A** | | **G** | **G** | **T** | **A** | **G** | **C** |  |
| Korea | SNU0044 | - | C | C | T | G | A |  | - | - | - | - | | - | - | - | - | - | - |  |
|  | SNU0046 | - | C | C | T | G | A |  | - | - | - | - | | - | - | - | - | - | - |  |
|  | iReo0309 | - | T | A | C | G | C |  | - | - | - | - | | - | - | - | - | - | - |  |
|  | ADL112770-ARV | - | - | - | - | - | - |  | A | T | A | A | | A | G | T | A | G | - |  |
|  | ADL112782-ARV | - | - | - | - | - | - |  | A | T | A | A | | A | G | T | A | G | - |  |
|  | ADL121187-ARV | - | - | - | - | - | - |  | A | T | A | A | | A | G | T | A | G | - |  |
|  | K738-14 | C | A | T | T | G | G |  | A | T | A | A | | A | G | T | A | G | C |  |

## Supplementary Table 5

| Group  (isolates) | ORFs |  | | p10 (amino acid) | | | | | | | | | | | | | | | | | | | | | | | | | | | | | | | | | |
| --- | --- | --- | --- | --- | --- | --- | --- | --- | --- | --- | --- | --- | --- | --- | --- | --- | --- | --- | --- | --- | --- | --- | --- | --- | --- | --- | --- | --- | --- | --- | --- | --- | --- | --- | --- | --- | --- |
|  | Position | | 44 | | 45 | | 46 | 47 | | 48 | 49 | | 50 | 51 | | | 52 | 53 | | | 54 | 55 | | | 56 | 57 | | | 58 | 59 | | | 60 | 61 | | 62 |  |
| Attenuated vaccine | av-S1133 | | Y | | L | | A | A | | G | G | | G | F | | | L | L | | | I | V | | | I | I | | | F | A | | | L | L | | Y |  |
| Commercial vaccine | S1133 | | Y | | L | | A | A | | G | G | | G | F | | | L | L | | | I | V | | | I | I | | | F | A | | | L | L | | Y |  |
| Vaccine break | GX110116 | | Y | | L | | A | A | | G | G | | G | F | | | L | L | | | I | V | | | I | I | | | F | A | | | L | L | | Y |  |
| Present study | A15-19 | | Y | | L | | A | A | | G | G | | G | F | | | L | L | | | I | V | | | I | I | | | F | A | | | L | L | | Y |  |
|  | A15-48 | | Y | | L | | A | A | | G | G | | G | F | | | L | L | | | I | V | | | I | I | | | F | A | | | L | L | | Y |  |
|  | A15-71 | | Y | | L | | A | A | | G | G | | G | F | | | L | L | | | I | V | | | I | I | | | F | A | | | L | L | | Y |  |
|  | A15-108 | | Y | | L | | A | A | | G | G | | G | F | | | L | L | | | I | V | | | I | I | | | F | A | | | L | L | | Y |  |
|  | A15-113 | | Y | | L | | A | A | | G | G | | G | F | | | L | L | | | I | V | | | I | I | | | F | A | | | L | L | | Y |  |
|  | A15-157 | | Y | | L | | A | A | | G | G | | G | F | | | L | L | | | I | V | | | I | I | | | F | A | | | L | L | | Y |  |
|  | A18-13 | | Y | | L | | A | A | | G | G | | G | F | | | L | L | | | I | V | | | I | I | | | F | A | | | L | L | | Y |  |
|  | A18-19 | | Y | | L | | A | A | | G | G | | G | F | | | L | L | | | I | V | | | I | I | | | F | A | | | L | L | | Y |  |
|  | A18-205 | | Y | | L | | A | A | | G | G | | G | F | | | L | L | | | I | V | | | I | I | | | F | A | | | L | L | | Y |  |
|  | A19-106 | | Y | | L | | A | A | | G | G | | G | F | | | L | L | | | I | V | | | I | I | | | F | A | | | L | L | | Y |  |
| Group  (isolates) | ORFs |  | | p17 (amino acid) | | | | | | | | | | | | | | | | | | | | | | | | | | | | | | | | | |
|  | Position | | 119 | | | 120 | | | 121 | | | 122 | | | | 123 | | | | 124 | | | | 125 | | | | 126 | | | | 127 | | | 128 | |  |
| Attenuated vaccine | av-S1133 | | I | | | A | | | A | | | K | | | R | | | | G | | | | R | | | | Q | | | | L | | | | D | |  |
| Commercial vaccine | S1133 | | I | | | A | | | A | | | K | | | R | | | | G | | | | R | | | | Q | | | | L | | | | D | |  |
| Vaccine break | GX110116 | | I | | | A | | | A | | | K | | | R | | | | G | | | | R | | | | Q | | | | L | | | | D | |  |
| Present study | A15-19 | | I | | | A | | | A | | | K | | | R | | | | G | | | | R | | | | Q | | | | L | | | | D | |  |
|  | A15-48 | | I | | | A | | | A | | | K | | | R | | | | G | | | | R | | | | Q | | | | L | | | | D | |  |
|  | A15-71 | | I | | | A | | | A | | | K | | | R | | | | G | | | | R | | | | Q | | | | L | | | | D | |  |
|  | A15-108 | | I | | | A | | | A | | | K | | | R | | | | G | | | | R | | | | Q | | | | L | | | | D | |  |
|  | A15-113 | | I | | | A | | | A | | | K | | | R | | | | G | | | | R | | | | Q | | | | L | | | | D | |  |
|  | A15-157 | | I | | | A | | | A | | | K | | | R | | | | G | | | | R | | | | Q | | | | L | | | | D | |  |
|  | A18-13 | | I | | | A | | | A | | | K | | | R | | | | G | | | | R | | | | Q | | | | L | | | | D | |  |
|  | A18-19 | | I | | | A | | | A | | | K | | | R | | | | G | | | | R | | | | Q | | | | L | | | | D | |  |
|  | A18-205 | | I | | | A | | | A | | | K | | | R | | | | G | | | | R | | | | Q | | | | L | | | | D | |  |
|  | A19-106 | | I | | | A | | | A | | | K | | | R | | | | G | | | | R | | | | Q | | | | L | | | | D | |  |

Deduced amino acid substitutions in the putative transmembrane domains of the p10 and nuclear localization signal motif in the p17
